# Normal values of neutrophil-to-lymphocyte ratio, lymphocyte-to-monocyte ratio and platelet-to-lymphocyte ratio among Iranian population: Results of Tabari cohort

**DOI:** 10.22088/cjim.10.3.320

**Published:** 2019

**Authors:** Mahmood Moosazadeh, Iradj Maleki, Reza Alizadeh-Navaei, Motahareh Kheradmand, Akbar Hedayatizadeh-Omran, Amir Shamshirian, Agil Barzegar

**Affiliations:** 1 Health Sciences Research Center, Addiction Institute, Mazandaran University of Medical Sciences, Sari, Iran; 2 Gut and Liver Research Center, Mazandaran University of Medical Sciences, Sari, Iran; 3 Gastrointestinal Cancer Research Center, Mazandaran University of Medical Sciences, Sari, Iran; 4 Department of Medical Laboratory Sciences, Student Research Committee, School of Allied Medical Sciences, Mazandaran University of Medical Sciences, Sari, Iran

**Keywords:** Normal values, Neutrophil-lymphocyte ratio, Lymphocyte-monocyte ratio, Platelet-lymphocyte ratio

## Abstract

**Background:**

Neutrophil-lymphocyte ratio (NLR), lymphocyte-monocyte ratio (LMR) and platelet-lymphocyte ratio (PLR) have a prognostic value in several types of diseases such as cancers and they vary in different races. So, we aimed to evaluate the normal range of these markers among healthy people to determine the normal value in Iranian population.

**Methods:**

In the present study, cross-sectional data of population-based cohort study named “Tabari cohort study” was utilized. In the first phase of Tabari cohort, 10255 participants aged 35-70 years from urban and rural areas of Sari, Mazandaran, Iran entered into the study. The study included a questionnaire survey and blood collection. Blood samples were collected after 12 hours fasting from all participants during the study. Hematological indices were measured for all samples using Celltac Alpha MEK-6510 K (Tokyo, Japan).

**Results:**

After sample exclusion, 2212 healthy subjects of Tabari's normal cohort population were investigated. The mean age of the samples was 47.9±9.29 years. The mean of NLR, LMR, PLR were 1.70±0.70, 11.15±3.14 and 117.05±47.73, respectively.

**Conclusion:**

Our investigation provides preliminary reference values for NLR, LMR, and PMR among Iranian population that can be used for disease progress in various clinical procedures.

Complete blood count (CBC) and differential leukocyte count called DIFF are the most common tests in clinical laboratories that can be measured by hematology auto-analyzers, (automated hematology analyzer) cost-effectively, rapidly and accurately (1). Outcomes of these tests as hematology values have been widely used in the study of the individuals’ health status, and according to these values, various blood and non-blood disorders can be evaluated (2). Laboratory results have little clinical importance unless they are described by providing a comparison between health status and disease. Therefore, the reference values are important in this regard, which provides the results of a seemingly healthy population. In addition, given that the values obtained in healthy subjects and patients can have significant overlaps, they should not be considered as an absolute indicator of health (3). Hematologic values are influenced by factors such as age, sex, race, nutrition, environment, above mean sea level, time and measurement methods. 

Therefore, when these are different in various populations, this diversity can lead to differences in reference values (2, 4). Since studies showed that alteration in the amount of peripheral blood cell can demonstrate the body inflammatory response, several investigations have indicated that blood-based indicators such as neutrophil to-lymphocyte ratio (NLR), lymphocyte to monocyte ratio (LMR) and platelet to lymphocyte ratio (PLR) can play a role as a potential prognostic indicator for various types of cancers (5-7).

Furthermore, in detail, NLR is known as an inflammatory marker as well as a significant prognostic factor for disorders such as cardiovascular diseases (8), different types of malignancies (9-12) and inflammatory bowel disease (IBD) (13). It is remarkable that inflammation as a biological response of the body against adverse stimuli plays a significant role in the development of cancer (14, 15). Systematic inflammation may have an effect on tumor microenvironment to the progression of the malignancy, which indicates a poor prognosis (16).

Moreover, previous studies showed that both LMR and PLR could play the same role as NLR in disorders such as gastric cancer (12) and urothelial carcinoma (17) respectively. Besides, several systematic review and meta-analyses have carried out to prove the prognostic effect of these indicators in solid tumors (18), breast neoplasm (19), stomach cancer (20), colorectal cancer (21), pulmonary cancer (22), etc.

In addition, although numerous studies have evaluated the impact of these indicators, normal values differ between them and it shows a considerable difference throughout the world among various ethnicities (23, 24). When these markers can be measured through a CBC test, which is a simple and cost-effective way, we can use these indicators in clinical process easily to detect a disorder or health follow-up in both the patients and the healthy population. Also, accordingly these indexes can be used as a prognostic factor, we need to know its normal values in each population to determine the cutoff points to use and compare them as normal values in later studies. 

Hence, as far as we know, there is no investigation regarding the normal range of NLR, LMR and PLR in Iranian population, we aimed to study the normal range of these markers and their relationships with demographic factors among healthy population contributing Tabari cohort in Iran.

## Methods

**Population and study design: **In the present study, we utilized cross-sectional data of population based cohort study named “Tabari cohort study”. In phase I of Tabari cohort, we registered 10255 participants aged between 35-70 from urban and rural areas of Sari city, Mazandaran, Iran (7012 urban and 3243 rural residents). It is worth mentioning that Tabari cohort is part of a nationwide cohort called “Prospective Epidemiological Research Studies in IRAN (PERSIAN) cohort” (25, 26). The study included a questionnaire survey and blood collection. Exclusion criteria of this study were as follows: participants with body mass index (BMI) less than 18 and greater than 30, individuals with at least one of the disorders including diabetes, hypertension, ischemic heart disease, stroke, myocardial infarction, renal failure, fatty liver, hepatitis B and C, asthma, thyroid disorders, kidney stone, gallstone, epilepsy, depression and other mental disorder, lupus erythematosus, multiple sclerosis (MS), any types of cancer and current smokers. 

**Body mass index: **BMI was measured by trained persons based on standardized methodology. Weight was measured using a calibrated balance scale of SECA 755 (SECA, Hamburg, Germany). To measure height, we used SECA 226 (SECA, Hamburg, Germany).

**Blood collection: **During the study, blood samples were collected after 12 hours fasting from all participants. Hematological indices were measured for all samples using Celltac Alpha MEK-6510 K (Tokyo, Japan). Lymphocyte, monocyte, neutrophil and platelet were reported as percentage and NLR, LMR and PLR were calculated.

**Statistical Analysis: **All statistical analyses were performed using Statistical Package for the Social Sciences (SPSS) software Version 24. Parameters such as percentage, mean and standard deviation (M±SD), median, range, minimum and maximum were used for data analysis. We used independent t-test and analysis of variance (ANOVA) to compare hematological indices and sex as well as residency and age groups, respectively.

## Results

In this study, 2212 subjects of Tabari's normal cohort population were investigated. The mean, standard deviation, median, minimum and maximum range of the samples’ age were 47.9, 9.29, 45, 35 and 70 years old, respectively. The subjects comprised 1120 (50.6%) females and the majority of the subjects were urban population (67%). The mean, standard deviation, average, and, minimum and maximum BMI of the subjects were 25.5, 2.77, 25.86, 18.07 and 29.99, respectively. The mean neutrophil-to-lymphocyte ratio in the whole population was 1.70±0.70 (Range: 8.38, Min: 0.23, Max: 8.61), mean lymphocyte-to-monocyte ratio was 11.15±3.14 (Range:23.21, Min:3.46, Max:26.67), and mean platelet-to-lymphocyte ratio was 117.05±47.73 (Range:93.60, Min:19.11, Max:1598.77). 

The mean lymphocyte-to-monocyte ratio was significantly higher in females than males (11.60±3.29 vs. 10.69±2.91; P<0.001), and it was higher in the urban population than the rural (11.38±3.24 vs. 10.68±2.88; P<0.001). In addition, the highest lymphocyte-to-monocyte ratio was observed in the age group under 40 years old (table 1). According to the results of Tukey's test, the differences observed in the mean lymphocyte-to-monocyte ratio among all age groups (except the age group of 40-49 years and 50-59 years) were statistically significant. 

The mean platelet-to-lymphocyte ratio was significantly higher in females than males (110.84±56.53 V.S 123.12±36.21; P<0.001). There was no significant difference between mean platelet-to-lymphocyte ratios among various age groups and rural or urban populations (table 2). Also, the mean neutrophil-to-lymphocyte ratio was not significantly different in terms of sex, age group, and residence (Table 3).

**Table 1:** The mean lymphocyte-monocyte ratio according to sex, age and area residence

**Variables**		**n**	**Mean±SD**	**95% CI mean**	**Min**	**Max**	**P-value**
Sex	Male	1092	10.69±2.91	10.52-10.86	3.46	26.50	<0.001
	Female	1120	11.60±3.29	11.41-11.79	4.12	26.67	
Age group	<40	642	11.73±3.54	11.45-12.00	5.95	26.67	<0.001
	40-49	747	11.14±2.91	10.94-11.35	4.35	23.12	
	50-59	543	10.91±3.11	10.65-11.18	3.46	26.50	
	>=60	280	10.29±2.52	9.99-10.59	3.98	19.55	
Residence	Urban	1482	11.38±3.24	11.22-11.55	3.46	26.67	<0.001
	Rural	730	10.68±2.88	10.47-10.89	3.98	26.50	

**Table 2:** The mean platelet-lymphocyte ratio according to sex, age and area residence

**Variables**		**n**	**Mean±SD**	**95% CI mean**	**Min**	**Max**	**P-value**
Sex	Male	1092	110.84±56.53	107.48-114.19	19.11	1598.77	<0.001
	Female	1120	123.12±36.21	120.99-125.24	33.21	322.59	
Age group	<40	642	114.71±35.89	111.92-117.49	41.71	322.58	0.358
	40-49	747	117.23±35.46	114.68-119.78	19.11	332.13	
	50-59	543	117.76±71.62	111.72-123.80	40.99	1598.77	
	>=60	280	120.59±41.71	115.69-125.50	33.21	398.66	
Residence	Urban	1482	115.90±51.89	113.26-118.55	40.99	1598.77	0.106
	Rural	730	119.39±37.82	116.64-122.14	19.11	336.52	

**Table 3:** The mean neutrophil-lymphocyte ratio according to sex, age and area residence

**Variables**		**n**	**Mean±SD**	**95% CI mean**	**Min**	**Max**	**P-value**
Sex	Male	1092	1.69±0.69	1.65-1.73	0.43	8.61	0.574
	Female	1120	1.71±0.71	1.67-1.75	0.23	7.63	
Age group	<40	642	1.72±0.75	1.66-1.78	0.43	7.63	0.162
	40-49	747	1.73±0.73	1.68-1.79	0.51	8.61	
	50-59	543	1.65±0.62	1.60-1.71	0.23	4.74	
	>=60	280	1.67±0.66	1.59-1.75	0.55	4.27	
Residence	Urban	1482	1.69±0.67	1.66-1.73	0.54	8.61	0.584
	Rural	730	1.71±0.76	1.66-1.77	0.23	7.63	

The highest mean lymphocyte-to-monocyte ratio was observed in male subjects in age group less than 40 years, and the highest platelet-to-lymphocyte ratio and neutrophil-to-lymphocyte ratio were observed in the 60-year-old age group and higher (Table 4). 

According to the results of Tukey's test, a significant difference was observed in the mean neutrophil-to-lymphocyte ratio in males in the age group of less than 40 and 50-59 (P=0.009), and, the age group of less than 40 with the 60-year-old age group and higher (P=0.026). The mean platelet-to-lymphocyte ratio in males showed a significant difference under the 40-year-old age group and 50-59 years old (P=0.022). 

In addition, table 4 shows that the highest lymphocyte-to-monocyte ratio, neutrophil-to-lymphocyte ratio, and platelet-to-lymphocyte ratio were observed in females under 40 years old. According to the results of Tukey's test, in females, a significant difference was observed in neutrophil-to-lymphocyte ratio in under 40-year-old age group and 50-59 years old (P<0.001), under 40-year-old age group and 60 years old (P=0.001), 40-49-year-old age group and 50-59 years old (P<0.001) and 40-49-year-old age group with 60 years old and higher (P=0.007). 

A significant difference was observed in the mean lymphocyte-to-monocyte ratio under the 40-year-old age group and 40-49 years old (P=0.004), 50-59 years old (P<0.001), 60 years old and higher (P<0.001) and between the 60-year-old age group and higher with 40-49 years old group (P=0.023). Additionally, a significant difference was observed in the mean platelet-to-lymphocyte ratio under the 40-year-old age group and 50-59-year-old group (P=0.002), and 50-59-year-old age group (P=0.003).

**Table 4:** The mean lymphocyte-monocyte ratio, platelet-lymphocyte ratio and neutrophil-lymphocyte ratio by sex and age group

**Variables**	**Male**					**Female**				
	**<40**	**40-49**	**50-59**	**>=60**	**P-value**	**<40**	**40-49**	**50-59**	**>=60**	**P-value**
LMR	10.88±3.03	10.75±2.79	10.75±3.09	10.17±2.55	0.079	12.27±3.73	11.49±2.97	11.16±3.14	10.49±2.48	<0.001
PLR	101.51±28.12	108.69±32.95	116.47±88.64	118.07±43.45	0.004	123.06±37.77	124.64±35.94	119.70±32.32	124.50±38.72	0.425
NLR	1.57±0.65	1.69±0.78	1.75±0.61	1.76±0.64	0.007	1.81±0.79	1.77±0.68	1.50±0.61	1.53±0.65	<0.001

## Discussion

Our investigation shows the first report of a cut-off values for NLR, LMR, and PLR in more than 2000 subjects among the Iranian population. There are few researches, which reported the normal values for these indicators. As far as we know, our study is the first study that made this comparison between these indicators by age, sex and residence among the healthy subjects of the Iranian population. In the present study, the mean NLR was 1.70±0.70 and it was not significantly different in terms of sex (P=0.574), age group (P=0.162), and place of residence (P=0.584). A few studies reported the NLR in healthy population. Azab et al.’s study in 2014, reported the mean NLR among 9427 samples in the “U.S. National Health and Nutrition Examination Survey”, in this study, the mean NLR was 2.15 (23) that was higher than our study, but in Forget et al’s study in 2017, the mean NLR was 1.65 (28), while in Lee et al.’s study, it was 1.65 (28) and in Kweon et al. study, was 1.53 (29), which were lower than our study. In our study, there were no significant NLR differences for sex (P=0.574), which were consistent with the results of Azab et al.’s (23) Kweon et al.’s and (29) Lee et al.’s (28) studies. In contrast to the present study, in Azab et al.’s (23) and Kweon et al.’s (29) studies, the relationship between NLR and age was reversed. In comparison to other studies, we found that the NLR of an Iranian population was more than the NLR of some other races. Besides, age distribution within the study populations may be one other possible causing this difference.

The mean LMR was 11.15±3.14 which was significantly higher in females (P<0.001), urban population (P<0.001) and age group under 40 years (P<0.001) compared to males, rural population and other age groups. The LMR among healthy population has been examined only in one study. This ratio in Lee et al. study was 5.31. In females, the LMR was more than in males (28) and in comparison with the present study, the LMR of an Iranian population was higher than the Korean population. These differences could be due to differences in the sampling approach except race and age distribution differences. The mean PLR was 117.05±47.73, which was significantly higher in females than males (P<0.001), but there was no significant difference between mean PLR among various age groups (P=0.358) and rural or urban population (P=0.106). The PLR among healthy population has been examined only in a few investigations. A study performed by Lee et al. (28) in 2018 reported the mean 132.4 for PLR, which was higher than our study. In Kweon et al.’s study (29), PLR was 121.07 which was lower than our study. It is remarkable that different types of secreted substances of platelets play mediatory role in coagulation, thrombosis, and inflammation process. For example, inflammatory states such as cancer effects on count and volume of platelet (28). 

In limitation, the calculation in the present study was included with single measures and variations could not be determined with time, but as a strength, the study was based on cohort study’s data and the selection of a normal population among this population, because the subjects with history of disease and main risk factor were excluded.

In conclusion, the present study provides preliminary reference value for NLR, LMR, and PMR among the Iranian population. The data indicate that various cutoff scores should be considered according to sex, age and residence, especially for LMR. These reference values can be used for disease progress in various clinical procedures.

## Funding:

This study was supported by one percent budget credit of Iranian Ministry of Health.

## Conflict of interests:

The authors declare that they have no competing interests.

## Availability of data and materials

The datasets analyzed during the current study are available.

## Authors' contributions

RAN designed the study. MM analyzed the data. MM, RAN, MK, and AS drafted the manuscript. All authors critically revised the manuscript, and read and approved the final version of the manuscript.

## Ethics approval and consent to participate

Tabari cohort has been approved by Mazandaran University of Medical Sciences Ethics Committee (IR.MAZUMS. REC.1395.2524).

## Patient consent for publication:

Not applicable.
